# Performance of low-cost noninvasive blood markers of liver cirrhosis in adults with chronic hepatitis B infection with and without comorbid alcohol use in Zambia: A cross-sectional hospital-based study

**DOI:** 10.1097/MD.0000000000048195

**Published:** 2026-04-03

**Authors:** Sydney Mpisa, Morris Kahere, Enock Syabbalo, Annie Kanunga, Michael Vinikoor, Edford Sinkala

**Affiliations:** a Department of Internal Medicine, University of Zambia School of Medicine, Lusaka, Zambia; b University Teaching Hospital, Lusaka, Zambia; c Posture and Back Pain Department, Reliable Spine, Scoliosis, Scotland, United Kingdom; d University of Alabama at Birmingham School of Medicine, Birmingham, AL.

**Keywords:** alcohol use, chronic hepatitis B, liver fibrosis, serum markers, transient elastography

## Abstract

Diagnosing liver cirrhosis in patients with chronic hepatitis B (CHB) remains challenging due to the infrequent use of biopsy. In low-and-middle-income countries, access to transient elastography (TE), a recommended noninvasive imaging modality for cirrhosis assessment, is limited. It is against this backdrop that we investigated the diagnostic performance of several low-cost and readily accessible blood-based liver fibrosis markers among patients with CHB infection in Zambia. We performed a hospital-based cross-sectional study in Lusaka, Zambia, among consecutive treatment naive adults presenting at the university teaching hospital with CHB mono-infection (i.e., human immunodeficiency virus-negative). The reference test for cirrhosis was TE of ≥9.6 kilopascals. Low-cost markers were the aspartate transaminase-to-platelet ratio index (APRI) at the recommended threshold >2, as well as lower proposed alternative thresholds for Africa, >0.5 and >0.65, aspartate transaminase/alanine transferase ratio, and fibrosis 4 index (FIB-4 index) >3.25. We evaluated the performance of each marker versus TE. In a secondary analysis, we evaluated marker performance in participants with current alcohol use versus lifetime abstinence. A total of 239 adults with HBV mono-infection were included in this analysis. The mean age was 34.7 years. About 22.2% (n = 53) reported current alcohol use. The prevalence of cirrhosis by TE was 16.3% (95% confidence interval: 11.87–21.63). The area under the receiver operating characteristic curve was 0.83, 0.80, 0.79 and 0.73 for FIB-4, APRI >0.5, APRI >0.65, and APRI >2, respectively. Virtually all indices performed less well in people with current alcohol use. These findings suggest the use of a lower APRI threshold in African settings and the use of the FIB-4 index for diagnosing cirrhosis among patients with CHB. The currently recommended APRI threshold may fail to identify some individuals with cirrhosis who would benefit from antiviral treatment. Clinicians using these markers should routinely screen for alcohol use and consider reevaluating cirrhosis status following reductions in alcohol consumption.

## 1. Introduction

According to the World Health Organisation (WHO) 2023 report, approximately 296 million individuals worldwide were affected with the hepatitis B virus in 2019.^[1]^ In sub-Saharan Africa (SSA), where hepatitis B is endemic, the overall sero-prevalence of hepatitis B remains as high as 6.1%.^[2]^ In Zambia, the sero-prevalence of Hepatitis B virus (HBV) was reported as 5.6% in 2016.^[3]^ This substantial prevalence of hepatitis B in SSA carries clinical ramifications, notably liver cirrhosis and hepatocellular carcinoma (HCC). Mozambique stands out as 1 with the highest HCC incidence, primarily linked to HBV.^[4]^

Once an individual is diagnosed with chronic HBV infection, indications for antiviral therapy are considered. A major factor in treatment decisions is the presence or absence of liver cirrhosis; however, diagnosing cirrhosis can be challenging since the “gold standard” is a liver biopsy, which is invasive and is associated with sampling errors.^[5]^ In 2015, based mostly on data from Asia and hepatitis C virus infections, the WHO recommended noninvasive markers of fibrosis, transient elastography, an imaging tool, and AST-to-platelet ratio index (APRI) score >2.^[6]^ While transient elastography (TE) is validated for diagnosing liver fibrosis,^[7]^ its limited availability in most hospitals underscores the need for affordable and accessible methods for liver fibrosis assessment in low-resource settings. Because of the low-cost and ability to be performed with existing equipment (unlike elastography), the APRI became widely adopted in low-and middle-income countries for HBV management; however, subsequent studies in Africa revealed that the cutoff threshold of >2.0 missed some patients requiring treatment.^[8]^ Achieving the WHO 2030 goals of eliminating HBV demands decentralizing and simplifying treatment for primary care physicians, and this calls for simpler ways of assessing liver fibrosis. More recently, APRI >0.65 was proposed based on analysis of African data.^[9]^

In this study, we aimed to assess the effect of low-cost noninvasive liver fibrosis markers (APRI, at current and proposed thresholds, fibrosis 4 index (FIB-4 index), and other similar indices) in forecasting liver fibrosis among patients with chronic HBV infection in SSA. First, we estimated the prevalence of liver cirrhosis among patients with chronic HBV mono-infection who are treatment naïve. We then assessed the degree to which the noninvasive liver fibrosis markers perform in diagnosing liver cirrhosis using transient elastography as the acceptable “gold standard” among patients with chronic HBV mono-infection. Because aspartate transaminase (AST) and alanine transferase (ALT) are nonspecific measures that can be increased with alcohol use, which is common in Zambia and the region, we also stratified analyses by alcohol use patterns.

## 2. Methods

### 2.1. Study design

This analytical and cross-sectional, hospital-based study was conducted from April 15, 2022 to January 24, 2023 at the University Teaching Hospital (UTH) in Lusaka, Zambia. The research involved reviewing medical records of adult male and female patients diagnosed with chronic hepatitis B (CHB) infection who provided consent and were attendees of the UTH hepatitis clinic. This study was conducted in accordance with the Declaration of Helsinki and was reported in compliance with the strengthening the reporting of observational studies in epidemiology checklist and explanation.

### 2.2. Study area and setting

The UTH, formerly Lusaka Hospital, is the largest public tertiary hospital in Lusaka, Zambia, with 1665 beds. The UTH hepatitis clinic is done weekly, staffed by trainees and consultant hepatologists and infectious disease specialists. Overall, nearly 500 persons with HBV are being followed up at our hepatitis clinic. UTH is a referral center and has enhanced access to HBV expert doctors and diagnostics; it has a large catchment population, including most of Lusaka.

### 2.3. Participants and eligibility criteria

Participants were included in this study if they were adults aged 18 years and above and had a positive result for Hepatitis B viral infection, defined by the presence of hepatitis B surface antigen (HBsAg) in blood. We excluded individuals who tested positive for human immune virus, those unwilling or unable to provide consent, those displaying signs of possible acute hepatitis (elevated ALT levels exceeding 3 times the upper limit of normal or jaundice), those with suspected HCC, individuals currently on antituberculosis drugs at the time of recruitment (that may alter liver transaminases), and those who had taken antiviral drugs for HBV infection for more than a month. These criteria were applied to ensure a targeted study population focused on CHB, while excluding individuals with potential confounding factors or acute conditions that could impact study outcomes.

### 2.4. Sampling and sample size estimation

Consecutive enrollment of all consenting participants with hepatitis B viral infection who were treatment naïve was adopted as it was deemed appropriate. Due to the cross-sectional nature of the study, the sample size was determined based on prevalence calculation. The minimum sample size was computed using the prevalence formula, which was estimated based on the following parameters: the required level of confidence, set at 95% (corresponding to a standard normal distribution value of 1.96); an assumed prevalence for liver fibrosis in the study setting, using 20% or 0.2 from a previous study done in Zambia^[10]^; and a desired degree of accuracy, specified at 5% or 0.05, representing the precision sought in the width of each arm of the confidence interval (CI). The required sample size for the study was 246.

### 2.5. Noninvasive serum markers of fibrosis

The use of serum markers of fibrosis has been an area of interest because liver cirrhosis requires histological confirmation, and this comes with its own challenges. APRI score is a ratio of the patient aminotransferase and platelet which is calculated as follows: **[(AST/upper limit of the normal AST range) X 100]/Platelet Count**. The calculated score can be used to diagnose liver cirrhosis, with different cutoff scores being suggested in the literature. WHO guidelines in 2015 adopted an APRI score >2 to diagnose liver cirrhosis. FIB-4 index (Fibrosis 4 index) is a test derived from the Apricot database. The FIB-4 index combines biochemical values (platelet count, ALT, and AST) and age as follows:

FIB-4 = Age (years) × AST (U/L)/ [PLT (10^9^/L) × ALT^1/2^ (U/L)]. A threshold value of 3.25 has a positive predictive value for the diagnosis of extended fibrosis of 65%.^[11]^ Of note, the FIB-4 does not have a specific threshold for cirrhosis, but we opted to evaluate it as it is low-cost and has potential for implementation in Africa. AST/ALT ratio is commonly used in alcoholic liver disease, where a ratio >2 is predictive,s but it has also been used to assess the extent of fibrosis in viral hepatitis. A ratio of AST/ALT above 1 has been used to diagnose liver cirrhosis, though its performance has been found to be unreliable.

### 2.6. Study instruments and data collection

Data were collected from the patients and patient medical records by a trained and experienced research assistant (a nurse), using a tool that was developed and pilot tested by the principal investigator prior to its final use. This tool collected information on participant demographic characteristics, medical history, alcohol history, and laboratory investigations, including HBV deoxyribonucleic acid (DNA), hepatitis B envelope antigen, and tests used in the low-cost fibrosis scores. The data collected within 30 days of the transient elastography were obtained for a valid comparison of the laboratory results and the TE values. The principal investigator checked/verified all the collected data to minimize bias and recording errors and to ensure accuracy and reliability of the data collection process.

### 2.7. Ethical consideration

Gatekeeper permission was sought from the participating institutions prior to seeking signed informed consent from the individual participants. The study was submitted to the University of Zambia Biomedical Research Ethics Committee and the National Health Research Authority for ethical approval. As it was an observational study, no interventions were instituted; patients continued to receive their usual care with no extra costs to the participants. Participation of all respondents in the study was strictly voluntary. Respect, dignity. and freedom of each individual participating in the study was granted. To guarantee the anonymity of each participant, the names of respondents, their addresses, or other identifying information were not included in the data collection. All participants were required to provide written informed consent; those who couldn’t consent for any reason were excluded from the study. The data was kept secure by the investigator, and a coding system was used to maintain confidentiality.

### 2.8. Statistical analysis

The data cleaning and analysis process utilized IBM statistical package for social sciences software version 27.0 for Windows (IBM Corp., Armonk). Descriptive statistics were employed, presenting frequencies and percentages for categorical variables, mean and standard deviation for age, and median with interquartile range for liver cirrhosis diagnostic markers. Alcohol use was categorized as never (i.e., lifetime abstinence), current use, or past use, because abstainers with past vs no lifetime alcohol use may have different degrees of liver disease. Sensitivity and specificity analyses were conducted using crosstabs to assess the performance of low-cost markers. The diagnostic performance of low-cost markers was expressed in terms of the sensitivity (se), specificity (sp), and area under receiver operating characteristic (AUROC). Optimised cutoffs for the low-cost markers and for liver cirrhosis were obtained by analyzing the AUROC at the maximum of total sensitivity and specificity. Dichotomous (1 for the presence of liver cirrhosis and 0 for NO cirrhosis) low-cost markers considered in the analysis were APRI > 0.65, APRI > 0.5, APRI > 2, FIB-4 index > 3.25, AST/ALT > 1, AST/ALT > 2, and Platelet count < 150. The TE score categories were defined as 0 (absence of cirrhosis, <9.6 kilopascals [kPa]) and 1 (presence of cirrhosis, 9.6 kPa or greater).^[12]^

## 3. Results

**Figure 1.**
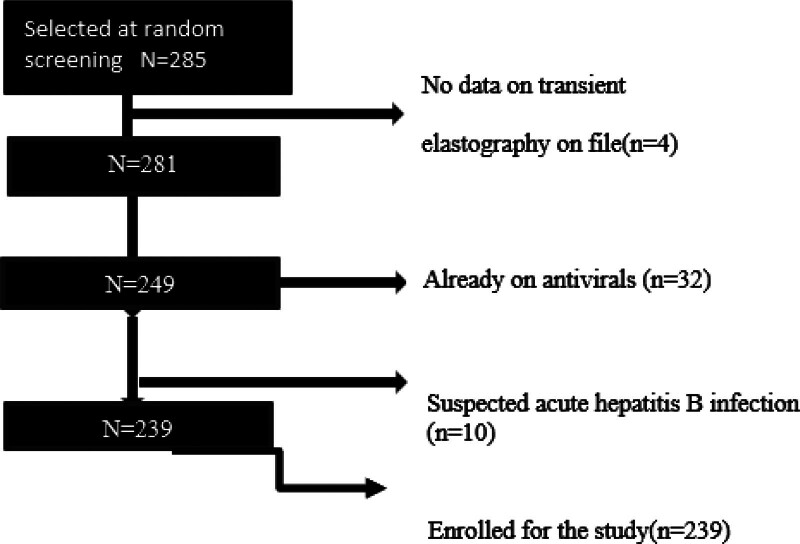
Recruitment process showing 285 participants selected at random screening whilst 4 participants got excluded because they didn’t have data on transient elastography. 32 participants were already taking antivirals thus got excluded and 10 participants got excluded because they had suspected acute hepatitis. A total of 239 participants got enrolled for the study.

A total of 285 patients were screened for inclusion in the analysis; 46 were excluded based on being on antiviral therapy for more than a month, nonavailability of TE results, and/or suspected acute HBV infection (Fig. 1), yielding an 83.9% eligibility rate. The male (n = 148, 61.9%) to female (n = 91, 38.1%) ratio was 3:2 with a mean age (±SD) of 34.7 (± 10.6) years. A little less than half (46.5%) of the participants had never drunk alcohol in their lives (i.e., lifetime abstainers) by report, with 23.0% and 30.4% being current and former drinkers, respectively. Variables with missing values are shown in Table 1.

**Table 1:** General characteristics of the participants analyzed (N = 239).

Variable			Value	Missing data
Age (years) mean ± SD			34.7 (10.6)	
Sex, N (%)				No missing values
		*Female*	91 (38.1)	
		*Male*	148 (61.9)	
Alcohol consumption pattern, N (%)				9
		*Current alcohol use*	53 (23.0)	
		*Former alcohol use*	70 (30.4)	
		*Lifetime abstainer*	107 (46.5)	
ALT (U/L) median (IQR)			25.0 (19–41)	6
AST (U/L) median (IQR)			28.5 (24–40.8)	3
Platelet count (/µL) median (IQR)			213.0 (164–260)	No missing values
Transient elastography score (kPa) median (IQR)			5.6 (4.5–7.4)	No missing values
HBV DNA results (%)			207 (86.6)	32 (13.4)
	HBV DNA VL < 2000 IU/mL		124 (59.9)	
	HBV DNA VL > 2000 IU/mL		83 (40.1)	
HB “e” antigen results (%)			101 (42.3)	138 (57.7)
	HB “e” antigen positive (%)		21 (20.8)	
	HB “e” antigen negative (%)		80 (79.2)	
APRI median (IQR)			0.4 (0.3–0.7)	3
AST/ALT ratio median (IQR)			1.2 (0.9–1.5)	8
FIB-4 median (IQR)			0.9 (0.6–1.5)	8

ALT = alanine aminotransferase, APRI = AST to platelet ration index, AST = aspartate aminotransferase, DNA = deoxyribonucleic acid, Fib-4 = fibrosis 4, HBV = hepatitis B virus, IQR = interquartile range, kPa = kilopascals, N = number, SD = standard deviation, VL = viral load.

Based on the TE cutoff of 9.6 kPa,^[12]^ the prevalence of liver cirrhosis in the analysis group was 16.3% (95% CI: 11.9–21.6). Among the 239 participants, 101 had hepatitis e antigen results, with 21 (20.8%) of those having positive results. A total of 207 participants had hepatitis B DNA viral load results, with 83 (40.1%) having a value more than 2000 IU/mL. We conducted a bivariate analysis (chi-square test) and it showed that APRI >0.65, APRI >0.5, APRI >2, FIB-4 index >3.25, platelet <150 and AST/ALT > 2 where significantly associated with cirrhosis except for the AST/ALT cutoff >1, *x*^2^ = 0.12, df = 1, *P* = .73. Among all the APRI thresholds, APRI cutoff 2 showed the greatest association (x^2^ = 97.31, df = 1, *P* < .001) with cirrhosis, and the association had a strong effect size (Cramer *V* = 0.64). FIB-4 index also had a strong association with cirrhosis (*x*^2^ = 88.73, df = 2, *P* < .001) with a strong effect size (Cramer *V* = 0.62).

### 3.1. Performance of low-cost markers to diagnose cirrhosis

**Figure 2.**
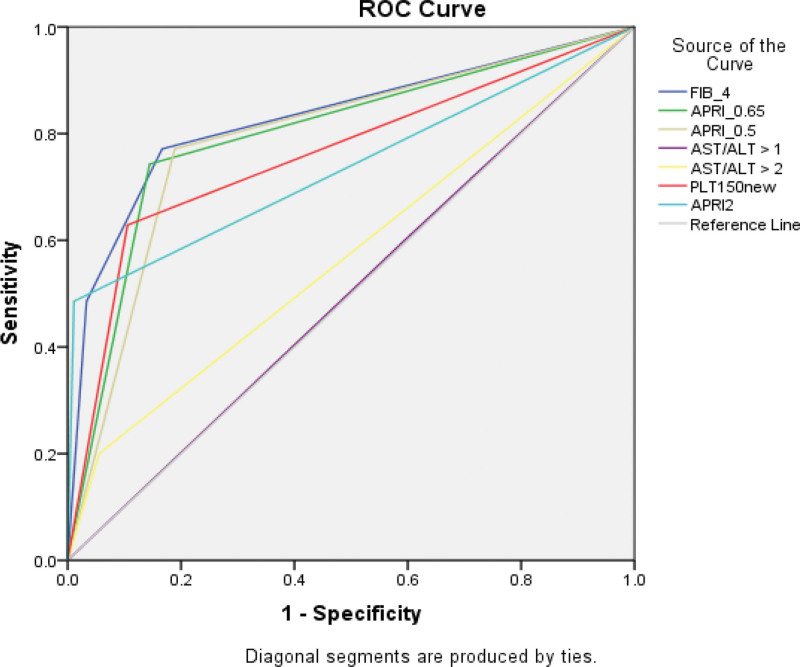
ROC (receiver operating characteristic) curve showing performance of different markers (APRI, FIB-4, AST/ALT, Platelet <150) in predicting cirrhosis based on transient elastography.

APRI > 0.65 had good sensitivity and specificity with values of 76.5% and 84.8%, respectively, whilst AST/ALT ratio >2 had the least sensitivity of 20%, showing a false negativity of 80% (Table 2). The current WHO recommended APRI cutoff 2 had good specificity (99%) but had very high false negativity 48.7% which means a number of patients can be missed, thus missing out on treatment. The FIB-4 index had the least false positivity at 3.1% and false negativity 20.5%, and when plotted on the AUC, it had the greatest AUROC, as shown in Figure 2. We also observed 66.7% sensitivity and 89.0% specificity for the simple platelet count at a threshold below 150.

**Table 2:** Performance of low-cost markers in diagnosis of liver cirrhosis.

Test performance	FIB-4 > 3.25	APRI > 0.5	APRI > 0.65	APRI > 2	AST/ALT > 1	AST/ALT > 2	Platelet < 150
Sensitivity (%)	51.3	79.5	76.9	51.3	40.9	20	66.7
Specificity (%)	83.3	80.7	84.8	99	62.2	94.8	89.0
False + ve (%)	3.1	19.3	15.2	1	59.1	5.2	11.0
False -ve (%)	20.5	20.5	23.1	48.7	37.8	80	33.3

FIB-4, APRI AST platelet ratio index.

ALT = alanine aminotransferase, APRI = AST to platelet ration index, AST = aspartate aminotransferase, Fib-4 = fibrosis 4.

### 3.2. Receiver operating characteristic analysis

Figure 2 and Table 3 delineate outcomes from a receiver operating characteristic curve (ROC) and an area under the curve (AUC) analysis assessing the diagnostic efficacy of APRI > 0.65, APRI > 0.5, APRI > 2, Platelet<150, AST/ALT > 1, FIB-4 index > 3.25, and AST/ALT > 2. AUC, a metric gauging diagnostic test performance, reveals values proximal to 1 signify superior efficacy. Results indicate that FIB-4 index exhibited the highest performance (AUC = 0.83, 95% CI: 0.74–0.91), slightly surpassing APRI > 0.5 (AUC = 0.80, 95% CI: 0.72–0.88), APRI > 0.65 (AUC = 0.78, 95% CI: 0.69–0.87) and platelets <150 (AUC = 0.76, 95% CI: 0.66–0.86). Conversely, AST/ALT ratios, both > 1 (AUC = 0.53, 95% CI: 0.44–0.63) and >2 (AUC = 0.53, 95% CI: 0.42–0.63), demonstrated relatively inferior performance. In summary, FIB-4 >3.25 emerges as the optimal performer, while APRI >0.5 cutoff exhibits marginally better efficacy than APRI >0.65 or platelets <150.

**Table 3:** Area under the ROC curve (AUROC).

Test result variables	AUROC	Standard error	*P*-value	Lower bound 95% CI	Upper bound 95% CI
APRI > 0.65	0.78	0.05	0.000	0.69	0.87
APRI > 0.5	0.80	0.04	0.000	0.72	0.88
AST/ALT > 1	0.53	0.05	0.507	0.44	0.63
FIB-4 > 3.25	**0.83**	0.04	0.000	0.74	0.91
AST/ALT > 2	0.53	0.05	0.628	0.42	0.63
Platelet < 150	0.76	0.05	0.000	0.66	0.86

ALT *=* alanine transaminase, APRI *=* aspartate platelet ratio index, AST *=* aspartate transaminase, AUROC *=* area under ROC, CI *=* confidence interval.

## 4. Discussion

At a specialized HBV clinic in Lusaka, Zambia, in a consecutive sample of treatment naive patients with chronic HBV mono-infection, 16.3% had cirrhosis based on TE. The most widely used (WHO recommended) low-cost index, APRI>2, performed less well (AUROC:0.73) compared to other APRI thresholds (APRI >0.5 and APRI>0.65, with AUROC of 0.80 and 0.78), respectively. FIB-4 had the highest AUROC (0.83) for cirrhosis, using a threshold of >3.25, which was previously proposed for fibrosis (i.e., pre-cirrhosis) in people with HCV in the United States guidelines.^[13]^ Finally, in a secondary analysis, we observed that across nearly all blood indices, accuracy was reduced in those individuals with comorbid alcohol use, as shown in Table 4. This project supports the implementation of HBV care in Africa and underscores the value of using local data to inform practice guidelines.

**Table 4:** Effect of alcohol on the performance of the noninvasive diagnostic tests.

Diagnostic test	Alcohol status	Sensitivity %	Specificity%	AUROC	95% CI
APRI > 0.65	Current	66.7	82	0.74	0.42–1.00
	Never	90.6	75	0.80	0.67–0.94
APRI > 0.5	Current	66.7	76.0	0.70	0.38–1.00
	Never	75.0	85.9	0.78	0.64–0.91
APRI > 2	Current	66.7	98	0.82	0.49–1.00
	Never	50.0	100	0.74	0.58–0.90
FIB-4 > 3.25	Current	0.0	77.6	0.70	0.38–1.00
	Never	55.0	86.9	0.80	0.67–0.95
Platelet < 150	Current	33.3	92.0	0.61	0.24–0.97
	Never	55.0	94.3	0.74	0.59–0.89

APRI *=* aspartate platelet ratio index, AUROC *=* area under ROC, CI *=* confidence interval, FIB-4 *=* Fibrosis 4 index.

In our evaluation of a range of low-cost markers to diagnose cirrhosis, to circumvent the need for elastography and biopsy, we found in Zambia that APRI >0.5, APRI >0.65, FIB-4, as well as a platelet <150 had the ability to diagnose cirrhosis. Notably, APRI score >2, based on WHO guidelines, was widely adopted, including in Zambia, but performed poorly in our study and 2 others in Ethiopia.^[14,8]^ This is clinically important as it could lead to missed opportunities to start treatment (i.e., too many false negatives), and this finding was also similar in our study, where we found APRI >2 with 48.7% false negativity, compared to 20.5% and 23.1% for APRI >0.5 and APRI >0.65, respectively. Our results agree with the latest findings from most studies, which show that lower thresholds like APRI >0.65 have better performance in predicting liver cirrhosis.^[15]^ A recent meta-analysis looked at the performance of APRI thresholds among patients with the CHB virus infection in SSA, and also found that APRI >0.65 had better performance in predicting cirrhosis compared to the WHO recommended APRI >2.^[9]^ In our study, APRI >0.5 had slightly better area under the curve than APRI >0.65 in the diagnosis of cirrhosis based on the TE cutoff 9.6 kPa, with areas of 0.80 and 0.78, respectively. Our study showed that FIB-4 index had the greatest AUC at 0.83, which is closest to 1 compared to the other noninvasive tests assessed in our study. This FIB-4 threshold >3.25 was proposed for fibrosis (i.e., pre-cirrhosis), but our data suggest it may have a role for cirrhosis detection too. Our results showed that a platelet cutoff of 150 was a good predictor of liver cirrhosis based on TE results. The Baveno VI consensus has adopted a platelet cutoff of 150 as a predictor for clinically significant portal hypertension.^[16]^ Based on the results from this study, we may adopt a platelet cutoff of 150 as another low-cost predictor of cirrhosis. The adoption of isolated low platelet count as a predictor of liver cirrhosis can be significant in remote areas where doing liver enzymes may be a challenge. The use of platelet count as a predictor of cirrhosis in chronic HBV infection should be applied in a clinical context since some regions in Zambia are endemic to schistosomiasis, which causes hypersplenism, thus causing a low platelet count in the absence of cirrhosis. Other common causes of a low platelet include such as malaria, bacterial and viral infections, should be considered.

Finally, we observed that across nearly all measures, current alcohol intake reduced the accuracy of the tests. Of the 239 enrolled participants, about half had a history of alcohol use, either current or past history; as such, the effect of alcohol on the noninvasive markers of fibrosis was an important research question. APRI thresholds >0.5 and >0.65 had reduced areas under the curve in those with current alcohol history, with values dropping from 0.80 to 0.74 for APRI >0.65 and 0.78 to 0.70 for APRI >0.5 when compared to lifetime abstainers. The trend was similar even for the FIB-4 index >3.25, which had an area of 0.70 in current alcohol drinkers compared to 0.81 in those who had never taken alcohol. This is an important finding as Africa has seen a rise in unhealthy alcohol use, and these data show that alcohol overlaps with the HBV epidemic in Southern Africa. We know that alcohol interferes with platelet production, and therefore, this could explain the poor performance of noninvasive markers in predicting cirrhosis.^[17]^ Alcohol also causes oxidative liver injury, resulting in elevation of the aminotransferases, and it increases the progression to liver cirrhosis among patients with CHB infection.^[18]^ A study done in 2006 showed APRI had low sensitivity and specificity for the diagnosis of significant fibrosis in patients with alcoholic liver disease, including patients who had hepatitis C.^[19]^ On the basis of our data, guidelines should strongly advocate that people with HBV be screened for alcohol use and that clinicians may consider reassessing for low-cost biomarkers of cirrhosis once alcohol use has reduced.

We also examined demographic factors associated with cirrhosis. Age of 40 years and above seems to be a risk factor for cirrhosis in patients with chronic HBV, according to a study done in France.^[20]^ Our study revealed a median age of 35; therefore, there is a need to be vigilant in detecting cirrhosis early in our patients. Our study had more males, almost twice as many as females, infected with chronic HBV, similar to the findings in South Africa.^[21]^ The sex disparity of HBV-related liver diseases has been noticed for a long time, which could be attributed to sex hormone effects, other than gender behaviors or environmental impact.^[22]^ Previous large cohort and 12-year follow-up studies showed that biological factors are more important than environmental or behavioral factors. The higher HBV in men indicates that females develop HBV antibodies faster than males, which helps them to clear HBsAg early. Additionally, the female sex hormone, estrogen, may be a protective factor for females, and androgen as a risk factor for men, contributing to higher HBV infections.^[23,24]^

### 4.1. Strength and limitation of this study

While the study had important strengths, including a moderate sample size of adults with chronic HBV mono-infection, use of transient elastography, and good clinical characterization, including related to alcohol use comorbidity and HBV DNA levels, it also had limitations. The cross-sectional study renders inferences on causality infeasible and limits follow-up for these patients. The study was a single site study done at a referral center, which is enriched with people with cirrhosis. Thus, the prevalence of cirrhosis in this sample is likely to be higher than in the general population. Also, alcohol was not objectively quantified or confirmed with a biomarker, which might bring some bias. For that reason, we excluded people with past use, whom we suspected may be falsely reporting abstinence, and also overcame the recall bias.

## 5. Conclusion

While low-cost liver fibrosis markers should be encouraged in Zambia and the regions, clinicians and policymakers should be well-versed in their strengths and limitations. We recommend adopting a lower APRI threshold (0.5 or 0.65) for Zambia and consideration of the FIB-4 index at >3.25 as predictors of liver fibrosis among CHB patients. Finally, alcohol screening and consideration should also be part of the HBV evaluation to determine when to start antiviral therapy in patients with CHB infection.

## Acknowledgments

All the patients who agreed to participate in the study.

## Author contributions

**Conceptualization:** Sydney Mpisa, Morris Kahere, Enock Syabbalo, Michael Vinikoor, Edford Sinkala.

**Data curation:** Sydney Mpisa, Annie Kanunga, Michael Vinikoor, Edford Sinkala.

**Formal analysis:** Sydney Mpisa, Morris Kahere, Michael Vinikoor, Edford Sinkala.

**Investigation:** Sydney Mpisa, Michael Vinikoor, Edford Sinkala.

**Methodology:** Sydney Mpisa, Morris Kahere, Edford Sinkala.

**Project administration:** Sydney Mpisa, Edford Sinkala.

**Validation:** Michael Vinikoor.

**Supervision:** Edford Sinkala.

**Writing – original draft:** Sydney Mpisa, Michael Vinikoor, Edford Sinkala.

**Writing – review & editing:** Sydney Mpisa, Morris Kahere, Enock Syabbalo, Michael Vinikoor, Edford Sinkala.
